# Still Large, but Narrowing: The Sizable Decline in Racial Neighborhood Inequality in Metropolitan America, 1980–2010

**DOI:** 10.1007/s13524-015-0447-5

**Published:** 2015-12-18

**Authors:** Glenn Firebaugh, Chad R. Farrell

**Affiliations:** Population Research Institute and Department of Sociology and Criminology, The Pennsylvania State University, 902 Oswald Tower, University Park, PA 16802 USA; Department of Sociology, Social Science Building 372, University of Alaska–Anchorage, 3211 Providence Drive, Anchorage, AK 99508 USA

**Keywords:** Neighborhood inequality, Racial inequality, Residential segregation, Neighborhood poverty, Concentrated disadvantage

## Abstract

Although residential segregation is known to have declined for some racial groups in America, much less is known about change in the relative socioeconomic quality of the neighborhoods where different racial and ethnic groups live. Using census data for 1980–2010, we find that the neighborhoods where whites and minorities reside have become more alike in terms of neighborhood poverty and median income, largely because whites now live in poorer neighborhoods and because African Americans live in less-poor neighborhoods. The narrowing of black-white neighborhood inequality since 1980 has been sizable, far exceeding the narrowing of Hispanic-white neighborhood inequality; nonetheless, despite blacks’ relative gains, the disparity in black-white neighborhood economic conditions remains very large. Asian Americans, on the other hand, now reside in neighborhoods that are economically similar to the neighborhoods where whites reside. Regression analyses reveal that racial neighborhood inequality declined the most in U.S. metropolitan areas where racial residential segregation declined the most.

## Introduction

The typical American city in the twenty-first century is characterized by residential inequality along race and class lines. As such, neighborhoods are where the nation’s racial and economic cleavages are most visibly “etched in place” (Sampson 2012:19). Neighborhood conditions historically have been particularly harsh for African Americans, a legacy of disadvantage that continues today as black-white inequality in neighborhood conditions notably exceeds black-white economic inequality at the individual or household level (Logan 2002, 2011). Rapid growth in Hispanic and Asian metropolitan populations—fueled by a combination of immigration, youthful age structures, and natural increase—has only intensified interest in neighborhood inequality. Our objective in this study is to investigate change since 1980 in the relative economic qualities of the neighborhoods where America’s four major racial/ethnic groups—Hispanics, non-Hispanic whites, blacks, and Asians—live.^1^

By examining change in racial inequality at the neighborhood level, this study fills an important gap in our knowledge about racial stratification in America. Despite broad consensus among social scientists that inequality in neighborhood environments produces inequality of opportunity for members of different racial and ethnic groups, we have surprisingly little quantitative evidence on how fast racial neighborhood inequality has been changing in the United States, and why. Only a few studies, such as Timberlake (2002), have examined change in the distribution of racial groups across all types of metropolitan neighborhoods (not just poor neighborhoods), and none of these studies have used the most statistically appropriate indexes of inequality, as we explain subsequently.

In this article, we describe change in racial neighborhood inequality in American metropolitan areas since 1980, the first year for which we have adequate census data for Hispanics. Our findings are based on a standard measure of inequality, the Gini index. Because the trends we uncover have gone largely unnoticed—and in some instances are contrary to conventional wisdom—we focus first on describing the contours of the change in racial neighborhood inequality. Our aim in the first part of this article is to present the most complete picture to date of change in the relative neighborhood environments of whites, blacks, Hispanics, and Asians over the past three decades in America. Then in the second part of this article we report findings from metropolitan fixed-effects regression models designed to account for the change we observe. Why has racial neighborhood inequality declined faster in some metropolitan areas than in others?

## Racial Neighborhood Inequality: What It Is and Why It Matters

By *racial neighborhood inequality*, we mean economic inequality: that is, disparity in the poverty rates and average incomes of the neighborhoods where different racial and ethnic groups live, reflecting the fact that racial and ethnic groups are unevenly distributed across rich and poor neighborhoods. In America, racial differences in household income account for only part of this disparity; many middle-class blacks, for example, reside in poor neighborhoods or in places surrounded by poor neighborhoods (Logan 2011; Patillo 1999; Sharkey 2014). It is important, then, to distinguish racial inequality at the neighborhood level from racial differences in poverty and income at the individual or household level.

It is also important to distinguish racial neighborhood inequality from racial residential segregation. *Residential segregation* refers to unevenness in the distribution of groups across neighborhoods. Although racial neighborhood inequality requires residential segregation—that is, racial groups can be unevenly distributed across rich and poor neighborhoods only if groups are unevenly distributed across neighborhoods—segregation does not preordain inequality.

Because studies of residential segregation show only that racial neighborhood inequality is possible—and not how large it is—the extensive literature on residential segregation alone is insufficient to draw conclusions about trends in racial neighborhood inequality. As Alba et al. (2008:14) noted, “While segregation indices can inform us about the extent to which members of different groups live in different neighborhoods, they cannot tell us directly about the ‘qualities’ of the neighborhoods in which group members reside.” If we agree that segregation matters largely because it contributes to the relative advantages and disadvantages of racial and ethnic groups (Cutler and Glaeser 1997; Massey and Denton 1993), then the analysis of residential segregation is insufficient. The crucial issue is racial differences in living conditions and life chances, not residential segregation *per se*.

Racial neighborhood inequality is particularly critical in America because poorer neighborhoods typically have significantly poorer social services, schools, and social environments, as well as less green space, higher crime rates, and more noise and congestion (Reardon and Bischoff 2011; Sampson et al. 1999). In terms of day-to-day existence, then, racial neighborhood inequality implies a lower quality of life for the average minority versus the average white, which might be one reason that African Americans in the United States report lower levels of happiness (Firebaugh and Schroeder 2009; Yang 2008). In addition, recent evidence suggests that prolonged exposure to poor neighborhood environments adversely affects one’s life chances along several domains (Crowder and South 2010; Sampson et al. 2008; Sharkey and Elwert 2011; Wodtke et al. 2011), and Sampson and Wilson (1995) argued that high-poverty environments encourage youth to pursue criminal careers. We should be concerned about racial neighborhood inequality, then, because it is consequential for the future as well as the present, serving as a wellspring for racial disparities in life chances now and in the days to come (Harding 2003; Sampson et al. 2008; Sharkey 2008, 2013).

The present study is particularly timely because racial neighborhood inequality in America—although much discussed—rarely is measured directly. Consequently, we have much evidence that minorities live in poorer neighborhoods in America but relatively imprecise estimates of the degree of the inequality, and even less precise estimates of how it has been changing.

## Evidence Bearing on Racial Neighborhood Inequality

Many studies have documented the overrepresentation of minorities in poor neighborhoods in America (Jargowsky 1996, 1997; Krivo et al. 1998; Massey 1990; Massey and Denton 1993). Supporting evidence comes from different disciplines and starting points. Research by urban planners and health policy researchers has found unevenness in the “geography of opportunity” of different racial and ethnic groups with respect to housing choice (de Souza Briggs 2005; Osypuk et al. 2009). Research by sociologists and other social scientists focusing on locational attainment (Alba and Logan 1991; Crowder and South 2005; Logan et al. 1996; Rosenbaum and Friedman 2006; Sampson and Sharkey 2008; South et al. 2005; Woldoff and Ovadia 2009), on economic segregation (Jargowsky 1996, 1997; Massey 1996; Reardon and Bischoff 2011; Wilson 1987), on concentrated disadvantage (Krivo et al. 1998; Massey and Denton 1993; Massey and Eggers 1990; Sampson et al. 2008), and on exposure to poverty in urban areas (de Souza Briggs and Keys 2009; Quillian 2003; Timberlake 2007; Timberlake and Iceland 2007) has also pointed to racial disparities in neighborhood environments.

Despite the enormity of this literature, we found only three studies (Osypuk et al. 2009; Timberlake 2002; Timberlake and Iceland 2007) that measured racial neighborhood inequality directly. Osypuk et al. (2009) found that racial neighborhood inequality is positively associated with residential segregation across the 100 largest U.S. metropolitan areas in 2000. Timberlake (2002) found much lower neighborhood inequality for Asians versus whites than for Hispanics versus whites or for blacks versus whites. In a follow-up study, Timberlake and Iceland (2007) found that although blacks remained the most residentially disadvantaged group in 2000, they exhibited the greatest relative improvement in neighborhood conditions from 1970 to 2000.^2^

However, none of these pioneering studies employed a standard measure of inequality. At a minimum, we want a measure of inequality that meets the usual requirements for inequality indexes (Allison 1978): the measure should be scale invariant, yielding the same value whether income units are given in dollars or in any other currency; should obey the principle of transfers so that transfers of income from richer to poorer units reduce the index, and transfers in the other direction increase the index; and should be *compositionally invariant*, meaning that the measure is not sensitive to changes in the relative sizes of groups—this property is important in the current study because of the rapidly changing racial composition of many areas in America (Lee et al. 2014). We also want an index that can measure segregation as well as inequality, permitting a transparent estimate of the effect of racial residential *segregation* on racial neighborhood *inequality* in our regression analyses. Finally, we want a measure that is commonly used so that we can easily compare racial neighborhood inequality with other types of inequality. The Gini index is the natural choice because it is commonly used, is scale invariant, obeys the principle of transfers, has properties that are well established, is compositionally invariant (Reardon and Firebaugh 2002), and measures segregation as well as inequality (Duncan and Duncan 1955; Hutchens 2004). In addition, Gini inequality can be depicted by Lorenz curves (Lorenz 1905). The inequality measures used in prior studies fail to satisfy all these criteria (see the appendix).

## Data

Using decennial census counts, we assess the residential circumstances of the four principal racial/ethnic groups in America: (1) non-Hispanic whites, (2) non-Hispanic blacks, (3) non-Hispanic Asians and Pacific Islanders, and (4) Hispanics of any race. Recent decades have seen momentous change in the composition of the U.S. population with respect to these groups. From 1980 to 2010, Hispanics increased from 6.4 % to 16.3 % of the U.S. population, and Asians increased from 1.5 % to 4.8 % (Gibson and Jung 2002; Humes et al. 2011). Although these changes are well documented, much less is known about what shifts have occurred in the economic environments where whites, blacks, Hispanics, and Asians live.

We use census-defined tracts as our measure of neighborhoods. Our tract-level data for 1980, 1990, 2000, and 2010 are based on summary files from the decennial U.S. Censuses, supplemented by the 2008–2012 American Community Surveys (ACS) for tract-level poverty rate and median income in 2010, as noted below. These tract data come from the Longitudinal Tract Data Base (Logan et al. 2014) and a GeoLytics database that provides estimates for untracted areas in 1980 (GeoLytics 2004). We include all metropolitan areas in the United States, thus capturing 77 % of the total U.S. population in 1980 and 84 % in 2010.^3^ To ensure that the trends we observe are based on a consistent set of boundaries, we standardize the pre-2010 census tracts to 2010 boundaries. We exclude tracts where more than 25 % of the residents live in group quarters (e.g., prisons), yielding a consistent set of 57,370 neighborhoods for each year. To test the robustness of our results, we repeated our analyses using 2000 census tract boundaries (from GeoLytics 2004, 2011a, b). Our conclusions are the same whether we use the 2000 or 2010 boundaries, as well as whether we include or exclude the areas that were not fully tracted in 1980.

To calculate racial neighborhood inequality, we need to know both the racial composition of neighborhoods and their economic conditions. Racial composition at the tract level is readily available from the decennial censuses. For neighborhood economic conditions, we use long-form census data for 1980, 1990, and 2000, and five-year ACS estimates centered on 2010 to derive two indicators: (1) tract poverty rate (percentage of individuals living below the poverty line) and (2) median household income. Both indicate the economic condition of neighborhoods, but they are not redundant (*r* = –.71 across tracts in 1980 and –.59 across tracts in 2010)—and, as Reardon and Bischoff (2011) noted, it is important to distinguish “segregation of affluence” from “segregation of poverty.” Poverty rate captures the lower end of the neighborhood income distribution, whereas median income reflects the level of middle and upper incomes as well. Thus, by comparing results for the two measures, we can determine whether racial differences in neighborhood environments are greater at the lower end of the income distribution or at the middle and upper ends of the distribution. Note also that the ACS data set we use to measure neighborhood poverty rate and median income in 2010 is based on surveys administered from 2008 through 2012, so our findings include the effects of the 2008 Great Recession on racial neighborhood inequality in America.

## Racial Neighborhood Inequality, 1980–2010

**Fig. 1:**
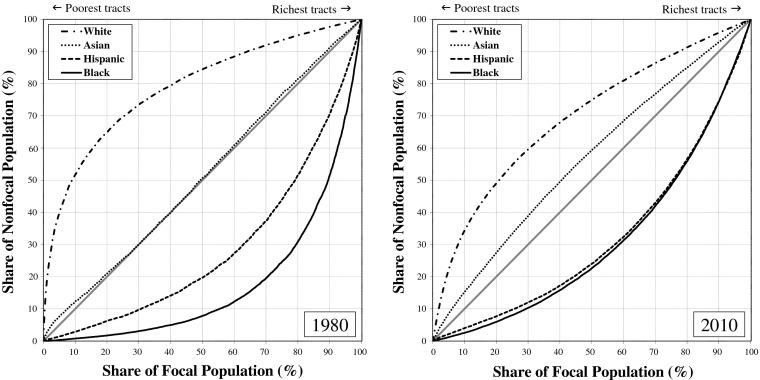
Narrowing of poverty-based racial neighborhood inequality in the United States, 1980–2010

**Fig. 2:**
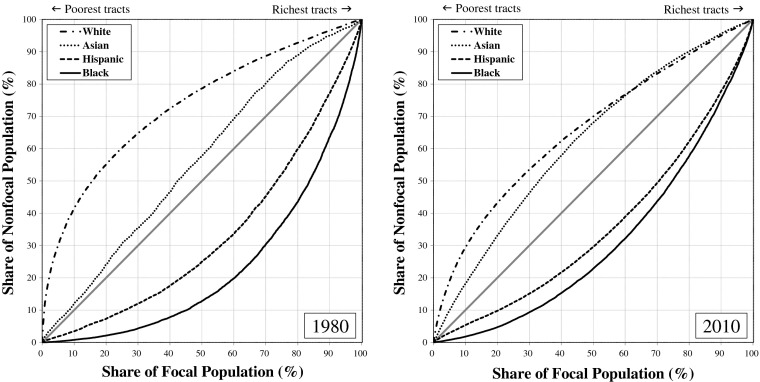
Narrowing of income-based racial neighborhood inequality in the United States, 1980–2010

Figure 1 (poverty-based inequality) and Fig. 2 (income-based inequality) display differences in the distributions of whites, African Americans, Hispanics, and Asian Americans across rich and poor neighborhoods in metropolitan America by depicting how much better off, or worse off, a group’s neighborhood conditions are compared with metropolitan residents who are not members of the group (the *nonfocal population*). If whites, blacks, Hispanics, and Asians were distributed proportionately over rich and poor neighborhoods, all four groups would lie on the line of equality in the graphs: that is, 10 % of each group would reside in the bottom 10 % of neighborhoods, 20 % of each group would reside in the bottom 20 % of neighborhoods, and so on. That is not what we find. In 1980 and in 2010, the curves for whites were well above the line of equality, reflecting whites’ greater concentration in richer-than-average neighborhoods; the curves for blacks and Hispanics, on the other hand, were well below the line, reflecting the greater concentration of blacks and Hispanics in poorer-than-average neighborhoods. In Fig. 1, the curve for Asians closely hugs the line of equality in 1980, indicating that Asians were the most typical Americans in 1980 in terms of the poverty rates of the neighborhoods where they resided. The curve for Asians lies above the line of equality in 2010, indicating that by 2010 Asians tended to live in neighborhoods with lower-than-average poverty rates.^4^

The greater the difference between the curves and the 45-degree line of equality, the greater the degree of racial neighborhood inequality. Clearly, whites tend to live in more economically advantaged neighborhoods than African Americans and Hispanics. Figures 1 and 2 nonetheless reveal a substantial narrowing of the racial neighborhood disparities since 1980. For both poverty-based and income-based neighborhood inequality, the curves for whites, blacks, and Hispanics were notably closer to the line of equality in 2010 than they were in 1980. For blacks and Hispanics, convergence to the line of equality represents reduction in relative neighborhood *disadvantage*. For whites, convergence to the line of equality represents reduction in relative neighborhood *advantage*. The neighborhoods where blacks and whites live and where Hispanics and whites live have become more alike economically since 1980.

Asians also are converging with whites, but the details differ. Rather than residing in typical American neighborhoods, as they did in 1980, Asian Americans now live in wealthier-than-average neighborhoods that are more similar to whites’ neighborhoods, particularly with respect to median household income. Thus instead of converging toward the line of equality, as is the case for the black and Hispanic curves, the Asian curve has expanded above the line, indicating an increase in residential advantage for Asians relative to other Americans.

**Fig. 3:**
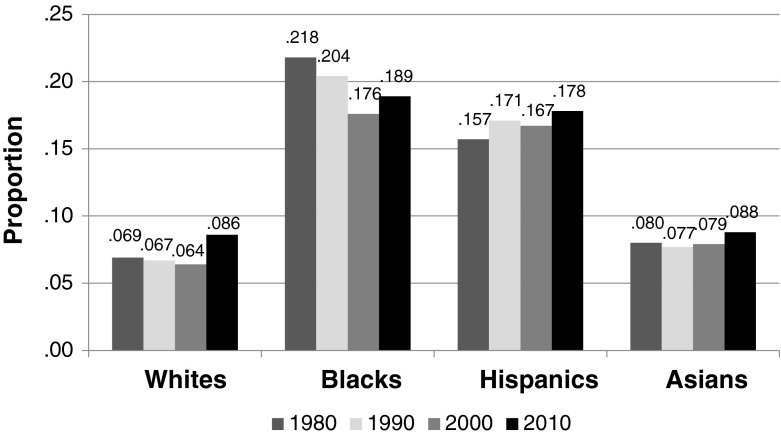
Proportion poor in the median neighborhood where whites, blacks, Hispanics and Asians live

In short, African Americans, Hispanics, and Asian Americans all gained on whites from 1980 to 2010, so change in racial neighborhood inequality over that period is a story of *declining white relative advantage*. The proximate causes are improved neighborhood conditions for blacks along with greater deterioration in neighborhood conditions for whites than for Hispanics and Asians. In 1980, one-half of blacks lived in neighborhoods where the poverty rate was greater than 21.8 %, and the other half where the rate was less than 21.8 % (Fig. 3). Contrast this with the situation for whites in 1980, when one-half lived in neighborhoods where the poverty rate was less than 6.9 %. The black-white difference narrowed from 1980 to 2010, with the median neighborhood poverty rate increasing to 8.6 % for whites and declining to 18.9 % for blacks. Although the difference that remains is pronounced, the point we want to underscore here is that the reduction in black-white neighborhood inequality is due both to higher poverty rates in the neighborhoods where whites live and to lower rates in the neighborhoods where blacks live.

The average neighborhood where Hispanics live had a higher poverty rate in 2010 than in 1980, but the percentage increase was less for Hispanics than for whites (13 % vs. 25 % for whites), resulting in a modest reduction in Hispanic-white neighborhood inequality. Median neighborhood poverty rate increased much less for Asians than for whites from 2000 to 2010, and it is noteworthy that by 2010 the median poverty rate was about the same in the neighborhoods of Asians and of whites (Fig. 3).

### When Did the Narrowing Occur?

**Fig. 4:**
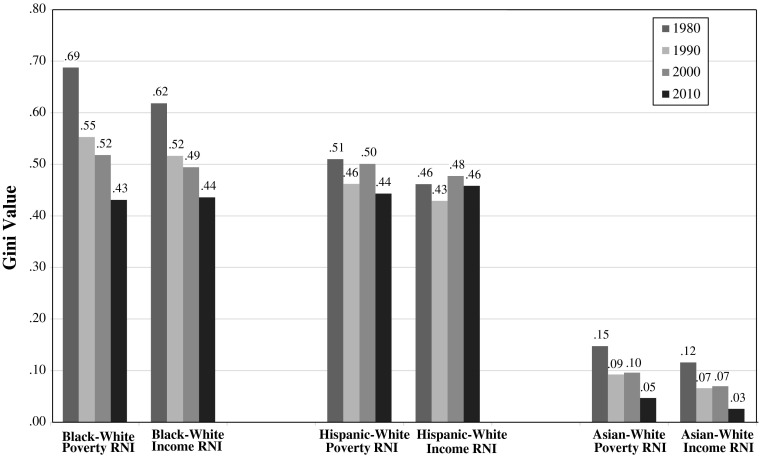
Change in black-white, Hispanic-white, and Asian-white neighborhood inequality in the average U.S. metropolitan area, 1980–2010

As Fig. 3 suggests, the narrowing of racial neighborhood inequality in America from 1980 to 2010 was due to the relative gains of minorities on whites, as neighborhood conditions improved for blacks and deteriorated faster for whites than for Hispanics and Asians. When did those gains occur? Figure 4 displays population-weighted average Gini coefficients for black-white, Hispanic-white, and Asian-white neighborhood inequality within American metropolitan areas in 1980, 1990, 2000, and 2010.^5^ Figure 4 shows that the difference between Asians and whites, which was modest in 1980, had largely disappeared by 2010. In the remainder of this article, then, we focus on disparities for African Americans and Hispanics.

Figure 4 reveals five critical facts about black-white and Hispanic-white neighborhood inequality in America. First, the degree of neighborhood inequality in 1980 was severe, especially for blacks (the black-white Gini was .69 based on poverty and .62 based on income).^6^ Second, black-white neighborhood inequality showed a sizable decline from 1980 to 2010; as measured by the Gini, poverty-based neighborhood inequality declined by nearly 40 %, and income-based neighborhood inequality declined by nearly 30 %, in the average metropolitan area over the period.

Third, for both blacks and Hispanics, poverty-based neighborhood inequality declined more than income-based inequality, indicating that the disparities in neighborhood conditions for blacks and Hispanics *vis-à-vis* whites narrowed more in terms of poverty rate than in terms of average neighborhood income. Apparently, convergence in the distribution of racial groups across poorer and richer neighborhoods was more pronounced at the lower end of the neighborhood income continuum.

**Fig. 5:**
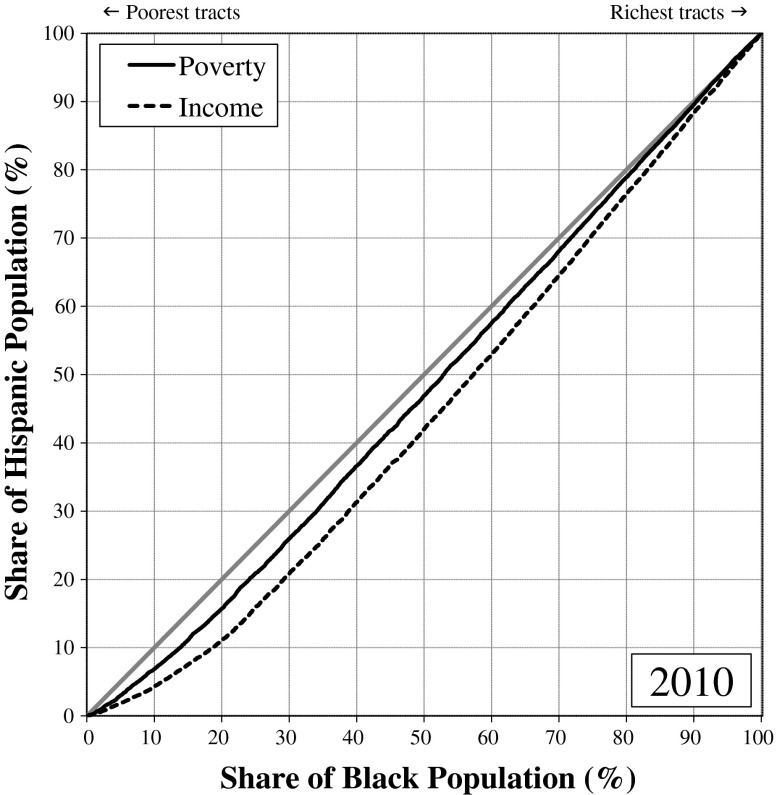
Lorenz curves showing little difference in the poverty rates and median incomes of the neighborhoods where Hispanics and blacks live

Fourth, black-white neighborhood inequality declined much faster than Hispanic-white inequality. For both poverty-based and income-based neighborhood inequality, the Gini coefficients in 1980 were one-third greater for blacks and whites than for Hispanics and whites. By 2010, black-white and Hispanic-white neighborhood inequality was virtually the same when we average the metro-level Gini values. This does not necessarily mean that Hispanics reside in neighborhoods as poor as those where blacks reside given that the comparison here is between blacks and whites who live in the same area with Hispanics and whites who live in the same metropolitan area, and Hispanics might be concentrated in metropolitan areas where whites tend to be more affluent than in the metropolitan areas where blacks live. When we adjust for regional differences in the affluence of whites by ignoring metropolitan boundaries, we find that, as of 2010, blacks still lived in poorer neighborhoods than Hispanics, but the differences are small (Fig. 5). Importantly, then, black-Hispanic neighborhood disparities have narrowed greatly since 1980. If these trends continue, Hispanics will reside in poorer neighborhoods than African Americans in the near future.

Finally, Fig. 4 shows that racial neighborhood inequality declined the most during the 1980s and the 2000s. This is true for African Americans, Hispanics, and Asian Americans. The narrowing of racial neighborhood inequality during the 2000s, the decade that included the Great Recession, is attributable largely to a near-doubling of the number of whites living in “poverty areas” (i.e., census tracts with poverty rates above 20 %).^7^ Between 2000 and 2010, the poverty rate for whites increased, and the percentage of poor whites living in poverty areas jumped from about 25 % to about 38 %. As a result, the number of whites living in poverty areas grew from 3.8 million to 7.5 million (Bishaw 2014). Because the percentage of poor nonwhites living in poverty areas increased much less over this period (Bishaw 2014), the residents of high poverty areas were more racially diverse by 2010 than in the past (Jargowsky 2014).

The increasing racial diversity of poor areas—poor whites living with poor minorities—is notable in light of recent evidence of a growing income divide among America’s neighborhoods (Massey et al. 2009; Reardon and Bischoff 2011). Perhaps the reduction in racial neighborhood inequality has been driven partly by the increasing economic segregation of America’s neighborhoods as middle-income blacks and Hispanics are less likely, and poor whites are more likely, to live in poor neighborhoods. To investigate this idea, among others, we regress our measures of racial neighborhood inequality at the metropolitan level on metropolitan characteristics that are likely to be associated with an area’s level of inequality. We now describe the model we use.

## Accounting for the Decline in Racial Neighborhood Inequality

### The Three Pillars of Racial Neighborhood Inequality

To reveal the characteristics of metropolitan areas that are associated with the greater decline in some metropolitan areas than in others, we perform a series of regression analyses based on metropolitan-level census data for 1980, 1990, 2000, and 2010. We begin by noting that racial neighborhood inequality in America rests on three pillars. The first pillar is difference in the racial composition of neighborhoods: *racial residential segregation*. The second pillar is difference in the average incomes in neighborhoods: *neighborhood income segregation*. Racial neighborhood economic inequality would be impossible if all neighborhoods were the same economically. The third pillar is *racial income inequality* at the household level. Blacks and Hispanics live in poorer neighborhoods in part because of their lower incomes (Clark 1986; Firebaugh and Farrell 2011).

We theorize that racial neighborhood inequality has changed because of change in each of the three pillars. Residential segregation has declined, although much faster for blacks and whites than for Hispanics and whites, which could account for the faster decline in black-white neighborhood inequality than in Hispanic-white neighborhood inequality. Racial income inequality was U-shaped over this period, with census data showing that median household income grew faster for Hispanics and blacks than for whites from 1980 to 2000 and then declined faster for Hispanics and blacks than for whites from 2000 to 2010. This U-shaped pattern in relative incomes for the United States as a whole masks differences across metropolitan areas, and we expect to find that racial neighborhood inequality declined faster in areas where minorities gained on whites economically than in areas where they did not.

The effect of change in neighborhood income segregation is harder to predict because of the possibility of compensating effects. After differences in household income are adjusted for, blacks and Hispanics tend to live in much poorer neighborhoods than whites. Sharkey (2014) found, for example, that the average black household with annual earnings of $100,000 lives in a more disadvantaged neighborhood than a white household with annual earnings of less than $30,000 (see also Logan 2002, 2011, 2013; Massey and Fischer 1999; Quillian 2012; Reardon et al. 2015; Woldoff and Ovadia 2009). Thus, in tightening the link between household income and neighborhood income, rising income segregation could “squeeze out” some of the neighborhood underplacement of minorities and overplacement of whites by reducing the proportion of middle-class blacks and Hispanics in poor neighborhoods and increasing the proportion of poor whites in poor neighborhoods.^8^ On the other hand, to the extent that rising income segregation is due to widening differences in average incomes across neighborhoods as opposed to narrowing income variance within neighborhoods, growing income segregation might increase racial neighborhood inequality by magnifying the effect of the income advantage of whites. If the two effects are offsetting, rising neighborhood income segregation might have little net effect on racial neighborhood inequality.

In short, we predict that racial neighborhood inequality declined the most in metropolitan areas where residential segregation declined the most and where minorities gained the most on whites economically. We speculate that the effect of change in income segregation will be modest because of the (possibly) opposing effects of declining within-neighborhood and increasing between-neighborhood income variance. To test these predictions, we estimated the following fixed-effects model for U.S. metropolitan areas in 1980, 1990, 2000, and 2010, where the subscripts *j* and *t* denote metropolitan area and time, respectively:

1 $$ RN{I}_{jt}={\upbeta}_0+{\upbeta}_1 ResSe{g}_{jt}+{\upbeta}_2 EconDisad{v}_{jt}+{\upbeta}_3 IncomeSe{g}_{jt}+Yea{r}_t+\updelta Siz{e}_{jt}+ Metr{o}_j+{\displaystyle \sum_k{\upgamma}_k{Z}_{jtk}+{\upvarepsilon}_{jt}}. $$

Equation (1) is designed to test our central idea that racial neighborhood inequality is rooted in racial segregation, racial inequality, and neighborhood income segregation. The equation states that racial neighborhood inequality (*RNI*) for a given metropolitan area is determined by the three pillars of racial neighborhood inequality—level of racial residential segregation, degree of minorities’ economic disadvantage, and income segregation of the area’s neighborhoods—as well as by other economic and demographic features of the area. The year dummy variables (*Year*) capture secular trends that are not accounted for by other variables in the model, and *Size* (measured by log of population) captures the effect of unaccounted-for differences between larger and smaller metropolitan areas.

By using a metropolitan fixed-effects model (*Metro*_*j*_), we eliminate the confounding effects of stable unmeasured characteristics of metropolitan areas that have constant effects over time (Allison 2005). These include potentially important confounders—such as topography, culture, and region of the country—that vary from area to area but are stable (or relatively so) over time. To control for pertinent characteristics that change over time, the *Z* term includes census measures of an area’s economic and demographic characteristics (e.g., overall poverty rate, proportion foreign-born). Because it is reasonable to assume that neighborhood change is more likely where many people are moving in or out and where new neighborhoods are sprouting up, we include variables designed to capture change in population composition or in housing stock (e.g., rate of new housing construction, proportion of labor force in the Armed Forces). A complete list of the control variables is found in Table 2 of the appendix.

### Measurement

Racial residential segregation (*ResSeg*) is the uneven distribution of racial groups across neighborhoods, and residential income segregation (*IncomeSeg*) is the uneven distribution of average household income across neighborhoods. We measure both using the Gini index.^9^ We use the minority/white poverty ratio to measure degree of minority economic disadvantage in our models of poverty-based racial neighborhood inequality and the ratio of white/minority average household income^10^ to measure minority disadvantage in our models of income-based racial neighborhood inequality. For both ratios, higher values indicate greater economic disadvantage for the minority group, implying a positive coefficient given that we expect racial neighborhood inequality to be the greatest in areas where minorities are the most greatly disadvantaged. Because the variables in our regressions are measured at the metropolitan level, we expect little measurement error in the 2010 ACS data, which are based on five years centered on 2010.

All our models include population size as a control variable. Controlling for population size nonetheless does not solve the issue of whether, for areas with the same total population, analyses of racial neighborhood inequality should give more weight to the area with more minority residents. Our solution is to compare results for alternative weighting schemes: (a) all 366 metropolitan areas, not weighted by the minority group of interest; (b) same as (a), but restricted to areas where the minority population of interest exceeds 10,000; and (c) all areas, weighted by size of the minority group of interest. We report results for each of the weighting schemes.

### Findings

Table 1 presents the estimated effects of the three pillar variables and population size, with and without the control variables. The findings are clear. Change in black-white and Hispanic-white neighborhood inequality from 1980 to 2010 was driven by change in residential segregation and by change in minority economic disadvantage. The effect of residential segregation is especially large, with regression coefficients approaching or exceeding 1.0. Because segregation and inequality are both measured in Gini units, this result indicates that a decline in racial neighborhood segregation is associated with a commensurate decline in racial neighborhood inequality. Also noteworthy is that the effects are as large for Hispanics as they are for African Americans.

**Table 1:** Metropolitan fixed-effects estimates^a^ of change in residential segregation, minority economic disadvantage, and neighborhood income segregation on change in racial neighborhood inequality, 1980–2010

	Poverty-Based Neighborhood Inequality		Income-Based Neighborhood Inequality	
	Base Model	Full Model	Base Model	Full Model
Black-White				
Residential black-white segregation				
All metropolitan areas	.824***	.878***	.903***	.952***
	(.066)	(.061)	(.074)	(.072)
All metropolitan areas, weighted by black population	.823***	.895***	.806***	.856***
	(.099)	(.076)	(.105)	(.103)
*N* > 10,000 blacks	1.038***	1.055***	.966***	.987***
	(.065)	(.067)	(.093)	(.101)
Residential income segregation				
All metropolitan areas	.108**	.074	.112**	.096*
	(.040)	(.038)	(.041)	(.040)
All metropolitan areas, weighted by black population	.101*	.025	.038	.009
	(.050)	(.028)	(.041)	(.031)
*N* > 10,000 blacks	.071*	.067*	–.005	.014
	(.031)	(.031)	(.027)	(.028)
Black economic disadvantage ratio				
All metropolitan areas	.035***	.035***	.019*	.016*
	(.007)	(.006)	(.009)	(.008)
All metropolitan areas, weighted by black population	.029***	.037***	.111***	.131***
	(.005)	(.005)	(.014)	(.018)
*N* > 10,000 blacks	.031***	.031***	.109***	.091***
	(.007)	(.006)	(.017)	(.021)
Population (logged)				
All metropolitan areas	–.058**	–.017	–.063*	–.027
	(.022)	(.031)	(.027)	(.034)
All metropolitan areas, weighted by black population	–.073***	.009	–.021	.008
	(.020)	(.016)	(.019)	(.020)
*N* > 10,000 blacks	–.065***	–.014	–.022	–.025
	(.017)	(.024)	(.020)	(.026)
Adjusted *R* ^2^				
All metropolitan areas	.44	.50	.41	.47
All metropolitan areas, weighted by black population	.85	.88	.68	.71
*N* > 10,000 blacks	.78	.80	.67	.70
Number of observations				
All metropolitan areas	1,461	1,461	1,462	1,462
*N* > 10,000 blacks	829	829	829	829
Hispanic-White				
Residential Hispanic-white segregation				
All metropolitan areas	.911***	.912***	.854***	.844***
	(.040)	(.041)	(.040)	(.042)
All metropolitan areas, weighted by Hispanic population	1.159***	1.104***	1.010***	1.102***
	(.050)	(.039)	(.092)	(.042)
*N* > 10,000 Hispanics	1.132***	1.088***	1.020***	.994***
	(.052)	(.046)	(.059)	(.053)
Residential income segregation				
All metropolitan areas	–.013	–.035	–.019	–.031
	(.030)	(.029)	(.031)	(.030)
All metropolitan areas, weighted by Hispanic population	.032	.025	–.025	.013
	(.042)	(.032)	(.048)	(.032)
*N* > 10,000 Hispanics	–.025	–.022	–.027	–.005
	(.031)	(.029)	(.031)	(.031)
Hispanic economic disadvantage ratio				
All metropolitan areas	.029***	.029***	.038***	.038***
	(.004)	(.004)	(.010)	(.010)
All metropolitan areas, weighted by Hispanic population	.008	.022***	.070*	.075***
	(.007)	(.005)	(.031)	(.018)
*N* > 10,000 Hispanics	.014**	.019***	.024	.024
	(.005)	(.005)	(.019)	(.017)
Population (logged)				
All metropolitan areas	–.065***	–.017	–.045**	–.020
	(.015)	(.022)	(.017)	(.023)
All metropolitan areas, weighted by Hispanic population	.009	–.030	.016	–.085***
	(.026)	(.021)	(.028)	(.023)
*N* > 10,000 Hispanics	–.055***	–.039	.024	–.075**
	(.015)	(.021)	(.019)	(.025)
Adjusted *R* ^2^				
All metropolitan areas	.67	.70	.65	.68
All metropolitan areas, weighted by Hispanic population	.79	.85	.71	.81
*N* > 10,000 Hispanics	.74	.78	.70	.74
Number of observations				
All metropolitan areas	1,464	1,464	1,464	1,464
*N* > 10,000 Hispanics	608	608	608	608

*Notes:* Standard errors, shown in parentheses, are adjusted for clustering. The base model includes dummy variables for year. To save space, we report those results—along with results for the additional 14 control variables in the full model—in Table 2 of the appendix. The *N* > 10,000 analysis is restricted to areas where the relevant minority population was greater than 10,000 (for at least two of the four census years, because metropolitan fixed-effects estimates are based on change within metropolitan areas). Racial neighborhood inequality, residential segregation, and neighborhood income segregation are measured using the Gini index. Minority economic disadvantage is measured as the minority/white ratio of poverty rates in the case of poverty-based neighborhood inequality and as the white/minority ratio of average incomes in the case of income-based neighborhood inequality.

^a^ Random-effects estimation yields similar results.

*p < .05; **p < .01; ***p < .001

The findings are also very robust. The coefficients for residential segregation and minority economic disadvantage change very little when we add control variables, indicating that the observed effects of changing residential segregation and changing minority disadvantage are not attributable to their correlations with changes in other metropolitan characteristics. Moreover, we find consistent results for residential segregation and minority disadvantage whether or not we weight areas by size of minority population: all 24 of the coefficients for residential segregation, and 21 of the 24 coefficients for minority disadvantage, are positive and statistically significant.

As predicted, then, racial neighborhood inequality has declined with declining racial segregation and with declining economic disadvantage for minority populations. With regard to residential income segregation—the theorized third pillar of racial neighborhood inequality—the coefficients are either positive or not statistically significant. Thus, we find no evidence for the “squeeze hypothesis” that rising income segregation in the United States has depressed racial neighborhood inequality by elevating the importance of income, and reducing the importance of race, as the basis for sorting households into neighborhoods.

Finally, it is noteworthy that a relatively simple model—the three pillar variables, population size, and dummy variables for year—does a good job of explaining why racial neighborhood inequality changed more in some metropolitan areas than in others. (Random-effects estimation (not shown) yields even stronger results.) The 14 control variables do not add much to the explained variance, nor do they materially affect the estimates for residential segregation and minority disadvantage, as we have noted. Our results for the control variables (see Table 2 in the appendix) indicate that, all other things being equal, poverty-based black-white neighborhood inequality diminished when proportion poor declined and when proportion Hispanic and proportion foreign-born grew. In the case of income-based black-white neighborhood inequality, only two control variables—proportion foreign-born and unemployment rate—had significant effects. For Hispanics, both poverty-based and income-based neighborhood inequality with whites tended to be reduced by growth in proportion homeowners and in proportion elderly and by reductions in average income, proportion black, and proportion suburban (other things being equal). In addition, poverty-based (but not income-based) Hispanic-white neighborhood inequality was affected by change in an area’s poverty rate, proportion Hispanic, and proportion foreign-born (see Table 2 in the appendix for complete results).

### Limitations

We began this study by examining trends in racial neighborhood inequality for all 366 U.S. metropolitan areas. We found that from 1980 to 2010, black-white neighborhood inequality declined much faster than Hispanic-white or Asian-white neighborhood inequality. Because neighborhood *segregation* also declined faster for blacks and whites than for the other dyads, this finding suggests that change in residential segregation played an important role in reducing racial neighborhood inequality. Our regression results indeed confirm a strong link between segregation and inequality. For both black-white inequality and Hispanic-white inequality, declines in segregation within a metropolitan area were associated with comparable declines in neighborhood inequality.

Although our findings reveal important new information about racial neighborhood inequality in America, there are limitations to what our data and model can investigate. In particular, by focusing on change in segregation and inequality within metropolitan areas, our fixed-effects analysis does not address the question of how migration across regions and metropolitan areas has affected racial neighborhood inequality for the nation as a whole—an important issue in light of the “new faces in new places” described in Massey (2008) and elsewhere (Hall 2013; Iceland 2009; Iceland et al. 2013). From the fixed-effects regressions, we observe a virtual one-to-one correspondence (in Gini units) between change in segregation and change in inequality within metropolitan areas. For metropolitan America as whole, however, the decline in Gini inequality exceeded the decline in Gini segregation. Presumably this is due, at least in part, to migration patterns across regions and metropolitan areas. We leave that question for future research.

Moreover, with tract-level data, we cannot directly examine change in the race-specific association between *household* socioeconomic status (SES) and neighborhood economic conditions. Because blacks and Hispanics very often live in much poorer neighborhoods than whites and Asians with similar incomes, we might expect racial neighborhood inequality to diminish as income becomes more decisive in determining one’s neighborhood. Yet we find no evidence that rising income segregation has reduced racial neighborhood inequality net of residential segregation and other neighborhood characteristics. If the growing income segregation of neighborhoods in America has narrowed income differences *within neighborhoods*—so that neighbors tend to be more similar in terms of income—then this narrowing apparently either has not materially boosted the proportion of affluent blacks and Hispanics in affluent neighborhoods or of poor whites and Asians in poor neighborhoods, or its effect has been muted by other consequences of rising income segregation. A full account of how rising income segregation affects racial neighborhood inequality must await more targeted investigations of the issue based on merged household- and neighborhood-level data.

## Discussion

A long-standing feature of American society is that whites, blacks, Hispanics, and Asians tend to live in different residential areas. Because this separation of groups remains high and is thought to have harmful consequences for minorities, residential segregation has been the subject of much research. However, separate is not necessarily unequal, and it is racial inequality in neighborhood environments, and the consequences of that inequality, that we should ultimately be most concerned about. It is well established that residence in a poor neighborhood is associated with heightened exposure to various social ills (such as crime) and reduced access to resources and services (such as good medical facilities and schools). As a result, racial inequality at the neighborhood level likely serves as the origin of many other types of racial disparities in American society by limiting the access of disadvantaged minorities to jobs, education, healthcare, and beneficial social networks. Although concerns about racial neighborhood inequality are not new (Du Bois 1899; Myrdal 1944), we know much more about change in residential segregation than we do about change in racial neighborhood inequality, in part because racial neighborhood inequality has not been measured with a standard inequality index.

Using the Gini index of inequality, we find that racial neighborhood inequality is very large and pervasive in America’s metropolitan areas. We also find that it has declined significantly over the three decades spanning 1980–2010 as whites have become less relatively advantaged, and blacks in particular have become less relatively disadvantaged. In 1980, blacks lived in the poorest neighborhoods by far, followed by Hispanics and then Asians. Over the next 30 years, neighborhood conditions improved for all three minority groups relative to whites, with blacks exhibiting the greatest improvement. By 2010, although black metropolitan residents continued to live in the most-disadvantaged neighborhoods, on average, neighborhood poverty rates and median incomes were much more similar for blacks and Hispanics than in 1980. Whites in 2010 were no longer unambiguously the most advantaged group in terms of residential economic environment: by 2010, Asians in many metropolitan areas resided in neighborhoods with lower poverty rates and higher median incomes than the neighborhoods where whites resided. Interestingly, income-based inequality declined more slowly than poverty-based inequality, perhaps indicating that the racial concentration of affluence is harder to reduce than the racial concentration of poverty.

A large portion of the 1980–2010 decline in black-white neighborhood inequality occurred between 2000 and 2010. At first blush, this recent reduction in black-white racial neighborhood inequality is surprising in light of evidence that the Great Recession had the most damaging effects on housing for African Americans (Hall et al. 2015; Kochhar et al. 2011; Rugh and Massey 2010). Consider, however, that the proportion of majority-black neighborhoods experiencing what Freeman and Cai (2015) called a “white invasion” (an increase in the white population exceeding 5 % of the total tract population) was greater from 2000 to 2010 than in the two previous decades combined (Freeman and Cai 2015). Moreover, virtually all the reduction in black hypersegregation over the 30-year period occurred since 2000, with the number of hypersegregated metropolitan areas in America declining by more than one-third from 2000 to 2010 (Massey and Tannen 2015). In the same vein, Owens (2012) found that from 1970 to 2009, the highest proportion of minority neighborhoods experienced “socioeconomic ascent” after 2000. Our finding of a substantial decline in black-white neighborhood inequality after 2000 is also less surprising in light of the near-doubling of the number of poor whites living in poverty areas from 2000 to 2010 (Bishaw 2014) combined with the growing number of middle-class African Americans who have escaped poor neighborhoods (Lacy 2007; Reardon and Bischoff 2011; Sharkey 2014).

After documenting the decline in black-white neighborhood inequality between 1980 and 2010, we used metropolitan fixed-effects regression to isolate the key drivers of the decline. The regression results contribute to knowledge about racial neighborhood inequality in three ways. First, the analyses reveal a very tight association between change in racial neighborhood inequality and change in racial residential segregation across U.S. metropolitan areas, with coefficients that approach or exceed 1.0 for the effect of segregation. As Massey and Denton (1993) insisted more than two decades ago, residential segregation serves as the foundation for black-white disparities in neighborhood economic conditions in America. Thus, when black-white segregation declines, we expect black-white neighborhood inequality to decline as well. Second, we discover that the association between neighborhood segregation and racial neighborhood inequality applies to neighborhood income as well as to neighborhood poverty. Prior research has focused on the effect of residential segregation on neighborhood poverty. Third, we find that the association between neighborhood segregation and neighborhood inequality is as strong for Hispanics and whites as it is for blacks and whites. Apparently, Hispanic-white neighborhood inequality is determined by the same forces as black-white neighborhood inequality, so Hispanic-white neighborhood inequality has declined more slowly than black-white neighborhood inequality largely because the decline in Hispanic-white segregation has lagged greatly behind the decline in black-white segregation.

### The Divergence That Remains

**Fig. 6:**
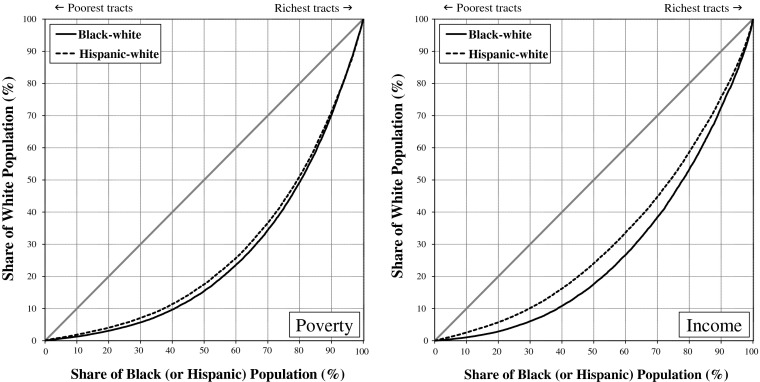
Lorenz curves for U.S. metropolitan areas showing black-white and Hispanic-white neighborhood inequality in 2010

Over the 1980–2010 period, black-white neighborhood inequality in the average metropolitan area declined by nearly 40 % based on neighborhood poverty and by nearly 30 % based on neighborhood median income. Despite this impressive reduction in black-white—and, to a lesser extent, in Hispanic-white—neighborhood inequality, large differences remain. To emphasize that point, we conclude by presenting the black-white and Hispanic-white Lorenz curves for neighborhood inequality in 2010. Figure 6 shows the Lorenz curves for poverty-based and income-based black-white and Hispanic-white neighborhood inequality in 2010 for U.S. metropolitan areas as a whole, ignoring metropolitan boundaries. (Unlike Figs. 1 and 2, where blacks are compared with nonblacks and Hispanics are compared with non-Hispanics, Fig. 6 compares blacks with whites and Hispanics with whites.) From these curves, we see the disparity that remains in the poverty rates and median household incomes of the neighborhoods where whites live versus where Hispanics live and where blacks live. For example, the poverty-based Lorenz curve for Hispanics and whites includes the point (50, 18), indicating that 82 % (that is, 100 % – 18 %) of whites live in neighborhoods where the poverty rate is lower than in the *average* (50th percentile) neighborhood where Hispanics live.

The disparity in neighborhood economic conditions is slightly greater for blacks versus whites: 84 % of whites live in neighborhoods where the poverty rate is lower than in the *average* neighborhood where blacks live. Even poor whites often fail to experience the levels of neighborhood disadvantage that are experienced by many affluent blacks (Massey and Brodmann 2014; Peterson and Krivo 2012). Racial disparities in neighborhood economic conditions remain large and consequential. Du Bois (1903) once pointed to the color line as the great problem of the twentieth century; despite three decades of change, that color line remains a defining problem for our fledgling century as well.

## Appendix: Measures of Inequality

Because inequality is a type of dispersion—a property of distributions as a whole—measures of inequality should take into account all points on a distribution. Measures that are based on a single point, or on parts of a distribution, fail to reflect intuition about inequality, such as the principle that inequality is reduced when income is transferred from a richer person to a poorer person (the transfer principle: Allison 1978; Cowell 2011). With respect to racial neighborhood inequality, the transfer principle means that inequality declines when a member of a disadvantaged group moves to a higher income neighborhood. The Gini measure we use is consistent with the transfer principle because it is based on the cumulative percentage of racial groups in neighborhoods. Hence, the Gini declines as the proportion of disadvantaged group members increases in higher-income neighborhoods.

The inequality measures used in prior studies do not necessarily capture the inequality-reducing or inequality-increasing effects of changes in the racial composition of rich and poor neighborhoods. Measures based on the interquartile range, for example, fail to capture the change in inequality resulting from change within quartiles. Osypuk et al. (2009) used two novel measures that reflect the amount of overlap of the distributions of the two groups being compared. Because the measures are based on a single point on the Lorenz curve of inequality, however, they fail to obey the transfer principle for inequality measures.^11^

The Timberlake (2002) and Timberlake and Iceland (2007) studies were based on the Index of Net Difference, or *p*(A > B) minus *p*(B > A), where *p*(A > B) is the probability that a randomly selected member of group A lives in a higher-ranking neighborhood than a randomly selected member of group B. Because it is unconventional, the use of this measure makes it difficult to gauge the size of racial neighborhood inequality relative to other types of inequality.

**Table 2:** Determinants of change in racial neighborhood inequality, 1980–2010: Results for the full model

	Poverty-Based Neighborhood Inequality	Income-Based Neighborhood Inequality
Black-White		
Residential black-white segregation	.895***	.856***
	(.076)	(.103)
Residential income segregation	.025	.009
	(.028)	(.031)
Black economic disadvantage ratio	.037***	.131***
	(.005)	(.018)
Population (logged)	.009	.008
	(.016)	(.020)
Year = 1990	–.004	–.004
	(.014)	(.014)
Year = 2000	.021	.021
	(.025)	(.025)
Year = 2010	.013	.013
	(.037)	(.037)
Average household income (in thousands of dollars)	–.0003	–.0001
	(.0006)	(.0006)
Proportion black	–.102	–.146
	(.134)	(.145)
Proportion Hispanic	–.228**	.025
	(.074)	(.098)
Proportion foreign-born	–.251***	–.120*
	(.043)	(.059)
Proportion poor	.383*	–.045
	(.166)	(.161)
Proportion homeowners	.153	–.304
	(.116)	(.154)
Proportion suburban	–.084	–.003
	(.071)	(.081)
Proportion vacant houses	.052	–.100
	(.102)	(.111)
Proportion new construction	–.049	.021
	(.050)	(.051)
Proportion age 65 or older	–.016	–.001
	(.308)	(.267)
Proportion female-headed households	.058	.087
	(.090)	(.071)
Proportion military	.031	.039
	(.116)	(.123)
Proportion manufacturing	–.112	–.053
	(.105)	(.093)
Proportion unemployed	–.340	–.389*
	(.172)	(.186)
Adjusted *R* ^2^	.88	.71
Hispanic-White		
Residential Hispanic-white segregation	1.104***	1.102***
	(.039)	(.042)
Residential income segregation	.025	.013
	(.032)	(.032)
Hispanic economic disadvantage ratio	.022***	.075***
	(.005)	(.018)
Population (logged)	–.030	–.085***
	(.021)	(.023)
Year = 1990	–.023*	–.008
	(.010)	(.012)
Year = 2000	–.038*	–.025
	(.017)	(.019)
Year = 2010	–.097***	–.076
	(.026)	(.029)
Average household income (in thousands of dollars)	.002***	.002***
	(.0003)	(.0004)
Proportion black	.470**	.536*
	(.177)	(.235)
Proportion Hispanic	–.236**	.041
	(.078)	(.090)
Proportion foreign-born	–.279***	–.112
	(.032)	(.075)
Proportion poor	.832***	.106
	(.131)	(.144)
Proportion homeowners	–.209*	–.509***
	(.100)	(.135)
Proportion suburban	.290***	.338**
	(.065)	(.097)
Proportion vacant houses	–.022	.066
	(.131)	(.112)
Proportion new construction	–.090	–.205**
	(.059)	(.071)
Proportion age 65 or older	–.694**	–1.085**
	(.259)	(.324)
Proportion female-headed households	–.026	–.027
	(.058)	(.083)
Proportion military	–.026	–.135
	(.209)	(.140)
Proportion manufacturing	.131	.256**
	(.082)	(.085)
Proportion unemployed	–.224	.177
	(.148)	(.136)
Adjusted *R* ^2^	.85	.81

*Notes:* Standard errors, shown in parentheses, are adjusted for clustering. Basic conclusions are the same regardless of the weighting scheme. Because it provides the best fit (largest *R* ^2^ values), we report coefficients for the model that includes all 366 metropolitan areas, weighted by population of the minority of interest. Our metropolitan control variables are constructed using U.S. Census data extracted from the Social Explorer (2014) website.

**p* < .05; ***p* < .01; ****p* < .001

## Footnotes

1 The four groups accounted for more than 97 % of the U.S. population in 2010. Although the term “Hispanic” is most often considered an ethnic designation, for simplicity we refer to whites, blacks, Hispanics, and Asians as racial groups. “Asian” includes Pacific Islanders. The excluded populations are non-Hispanics who indicated that they are of more than one race (1.9 % of the U.S. population in 2010), American Indian or Alaska natives (0.7 %), and non-Hispanics who indicated “some other race” (0.2 %).

2 Timberlake and Iceland (2007) expanded the Timberlake (2002) analysis by adding data for 1970 and 2000 and using a regression model to determine the predictors of racial neighborhood inequality in 2000 and its change from 1970 to 2000.

3 As defined by the U.S. Office of Management and Budget (OMB), a metropolitan area contains a core urban area population of 50,000 or more. It is comprised of the county or counties in which the core is located as well as any adjacent counties that are socially and economically integrated with the core. We use the OMB December 2009 update of metropolitan definitions: http://www.census.gov/population/metro/files/lists/2009/List1.txt.

4 We used the logic of Lorenz curves (Lorenz 1905) to create Figs. 1 and 2. First we arranged the 57,370 metropolitan census tracts from poorest to richest and calculated the cumulative percentage of our four focal populations (whites, blacks, Hispanics, and Asians) and their complements (e.g., nonwhites) across the tracts. Then we plotted the results in a graph where the *x*-axis is cumulative percentage of the focal group (e.g., whites) and the *y*-axis is the cumulative percentage of the remainder of the metropolitan population, the non-focal population (e.g., nonwhites). In Fig. 1, then, the point (50, 8) on the curve for blacks in 1980 tells us that only 8 % of nonblacks lived in neighborhoods where the poverty rate was as high as, or higher than, it was in the average (50th percentile) neighborhood where blacks lived in 1980. Thus 92 % of nonblack Americans lived in neighborhoods where the poverty rate was lower than in the *average neighborhood* where blacks lived*.* Likewise, the point (9, 50) on the curve for whites tells us that only 9 % of whites lived where the poverty rate was as high as, or higher than, it was in the average neighborhood where nonwhites lived, so 91 % of whites lived where poverty was lower than in the average neighborhood where nonwhites lived. In short, the racial disparities in neighborhood conditions in 1980 were huge.

5 The Gini coefficient is a function of the area between the group curves and the line of equality (Gini 1912, 1921). Gini coefficients range from 0 to 1 in absolute value, with a value of 0 indicating no difference in the economic conditions of neighborhoods for the groups being compared, and a value of 1 indicating that all members of the advantaged group live in richer neighborhoods than all members of the disadvantaged group (complete neighborhood inequality). The Gini values in Fig. 4 are weighted by size of the minority population so that metropolitan areas with larger minority populations are given more weight.

6 As a point of reference, the estimated Gini values for income inequality in the United States are .51 for pretax income and .39 for post-tax, post-transfer income in 2012 (OECD 2014).

7 The decline is not due to measurement error in the ACS data for 2010. Our cross checks of similar data in the ACS and decennial census data revealed virtually identical results. For example, the decennial census and the ACS both collected data on homeownership at the tract level, and the Gini for black versus nonblack ownership across tracts was .312 based on the decennial census and .310 based on ACS data.

8 Quillian (2012) found that concentrated poverty among blacks and Hispanics in America is due largely to the poverty of their other-race neighbors: for example, middle-class blacks living beside poor nonblacks. Rising income segregation could reduce that cross-race effect.

9 We use Duncan and Duncan’s (1955) formula for calculating the Gini based on the difference in two cumulative distributions. For residential income segregation, we calculate the Gini from the cumulative distributions of population and income across the 57,370 neighborhoods, where the neighborhoods are arranged from poorest to richest on the basis of median income. In the case of residential segregation from whites, we order neighborhoods on the basis of minority composition (e.g., from neighborhoods with highest % black to those with lowest % black), and calculate the Gini from the cumulative distribution of the minority group across the neighborhoods compared with the cumulative distribution of whites across the neighborhoods.

10 We substitute family income for household income in 1980 because household income was not broken down by race in the 1980 census. The correlation of (overall) average family income with average household income was .98 across metropolitan areas in 1980.
